# The Role of Mitochondrial Quality Control in Anthracycline-Induced Cardiotoxicity: From Bench to Bedside

**DOI:** 10.1155/2022/3659278

**Published:** 2022-09-21

**Authors:** Yukun Li, Rong Lin, Xiaodong Peng, Xuesi Wang, Xinmeng Liu, Linling Li, Rong Bai, Songnan Wen, Yanfei Ruan, Xing Chang, Ribo Tang, Nian Liu

**Affiliations:** ^1^Department of Cardiology, Beijing Anzhen Hospital, Capital Medical University, Beijing 100012, China; ^2^National Clinical Research Center for Cardiovascular Diseases, Beijing 100012, China; ^3^North China Medical & Health Group Xingtai General Hospital, Xingtai 054000, China; ^4^Department of Cardiology, Beijing Chuiyangliu Hospital, Beijing 100012, China; ^5^Banner University Medical Center Phoenix, College of Medicine University of Arizona Phoenix, Arizona 85123, USA; ^6^Guanganmen Hospital, China Academy of Chinese Medical Sciences, Beijing 100053, China

## Abstract

Cardiotoxicity is the major side effect of anthracyclines (doxorubicin, daunorubicin, epirubicin, and idarubicin), though being the most commonly used chemotherapy drugs and the mainstay of therapy in solid and hematological neoplasms. Advances in the field of cardio-oncology have expanded our understanding of the molecular mechanisms underlying anthracycline-induced cardiotoxicity (AIC). AIC has a complex pathogenesis that includes a variety of aspects such as oxidative stress, autophagy, and inflammation. Emerging evidence has strongly suggested that the loss of mitochondrial quality control (MQC) plays an important role in the progression of AIC. Mitochondria are vital organelles in the cardiomyocytes that serve as the key regulators of reactive oxygen species (ROS) production, energy metabolism, cell death, and calcium buffering. However, as mitochondria are susceptible to damage, the MQC system, including mitochondrial dynamics (fusion/fission), mitophagy, mitochondrial biogenesis, and mitochondrial protein quality control, appears to be crucial in maintaining mitochondrial homeostasis. In this review, we summarize current evidence on the role of MQC in the pathogenesis of AIC and highlight the therapeutic potential of restoring the cardiomyocyte MQC system in the prevention and intervention of AIC.

## 1. Introduction

Mitochondria are essential intracellular organelles of cardiomyocytes and are involved in the regulation of a wide array of biological processes that govern cell division, growth, and death, including lipid metabolism, energy production, regulation of redox homeostasis, and cellular calcium buffering [1–4]. Mitochondria produce the majority of adenosine triphosphate (ATP) via the electron transport chain (ETC) located on the inner mitochondrial membrane (IMM). Normal cardiac contractile function is sustained by ATP production via mitochondrial oxidative phosphorylation (OXPHOS).

Mitochondria are most abundant in the heart as it is the most metabolically demanding organ in the human body [5, 6]. It is estimated that cardiac mitochondria supply ATP to the human heart at an incredible rate of 6 kg/day [7]. In addition to the energy supply, mitochondria can regulate a series of biological processes, such as cell growth, ion transport, signal transduction, and gene expression [8]. Changes in mitochondrial morphology and function can be seen as a microcosm of cardiomyocyte response to a wide range of external stress [9, 10]. Thus, an imbalance in mitochondrial homeostasis is strongly correlated with cardiac dysfunction owing to the corresponding pathological mechanisms such as oxidative stress, metabolic dysregulation, inflammation, disrupted calcium homeostasis, and autophagy [11–13]. Moreover, excessive reactive oxygen species (ROS) production under pathological conditions can induce several forms of cell death, such as necroptosis, pyroptosis, and ferroptosis and disrupt myocardial proliferation and differentiation [14]. Thus, it is not difficult to understand that the physiological functions of the heart depend strongly on the maintenance of mitochondrial homeostasis.

Mitochondria have evolved a suite of adaptation systems to preserve their function in the face of external stress, including morphological changes, enhanced biogenesis, and accelerated self-renewal. These homeostatic responses are mechanistically generated by a cascade of molecular and cellular events, which are collectively termed as the mitochondrial quality control (MQC) system [15, 16]. Dysfunction in these adaptive responses may inevitably induce mitochondria-dependent cell death [17, 18]. Therefore, the MQC system, which refers to a series of mechanisms evolved to maintain mitochondrial integrity and function, is essential for cardiomyocytes under stress or disease states.

The MQC system exerts effects at both molecular and organelle levels. The former primarily includes the regulation of mitochondrial chaperones and ATP-dependent proteases, as well as the mitochondrial unfolded protein response (UPRmt), which are collectively termed protein quality control mechanisms. The latter organelle quality control mechanisms involve mitochondrial dynamics (fusion/fission), mitophagy, and mitochondrial biogenesis. When the MQC system fails to play its regulatory role [14], it may contribute to the incidence of many diseases such as kidney injury [14], pulmonary fibrosis [19], cardiac ischemia/reperfusion (I/R) injury, and so on [10, 17, 20].

Anthracycline-induced cardiotoxicity (AIC) remains the most widely studied antitumor drug-induced toxicity. Failure of MQC plays a key role in the onset and progression of AIC, because anthracycline can disrupt mitochondrial homeostasis and lead to mitochondrial dysfunction. ROS overgeneration is the most widely accepted theory proposed to explain the pathogenesis of AIC. An increasing amount of ROS can induce mitochondrial dysregulation by depolarizing the mitochondrial membrane, which further contributes to increased mito-ROS production and forms a feedback loop [21–23]. Therapies targeting the MQC system have emerged as promising strategies for the prevention and treatment of AIC. In this review, we provide a brief description of AIC as well as the current findings pertaining to the molecular basis of MQC, which includes the mechanistic signaling pathways, physiological functions, and pathological relevance of AIC. We then summarize the promising MQC-targeting therapeutic strategies that are being developed for AIC.

## 2. Anthracycline-Induced Cardiotoxicity

The research and development of antitumor drugs have significantly improved the clinical survival outcomes of patients with cancer. Anthracycline is a widely used chemotherapeutic agent for many types of malignancies, including lung cancer, breast cancer, and hematological malignancies [24, 25]. With the improvement in progression-free survival and overall survival, clinicians pay more attention to adverse events after long-term administration of anthracycline [26, 27].

The main adverse drug reactions (ADRs) associated with anthracycline include cardiotoxicity, bone marrow suppression, gastrointestinal reactions, and liver/kidney damage. Among them, AIC is considered the most prominent and serious adverse effect [28, 29]. Moreover, AIC became more significant with increasing doses, exhibiting dose-dependent myocardial damage. Research in related areas has shown that the incidence of AIC can reach nearly 26% and 48% when the cumulative doses reach 550 mg/m^2^ and 700 mg/m^2^, respectively [30].

AIC can be subdivided into two categories: acute and chronic [31]. Acute AIC can occur during anthracycline treatment for several days to weeks after antitumor treatment. This type of cardiotoxicity affects almost 30% of the patients. It is characterized by abnormal electrocardiogram (ECG) changes, such as decreased QRS voltage, atypical ST changes, prolonged QT interval, and different types of heart block. Dose modification or even cessation of anthracycline treatment may be needed in this situation [32, 33]. Chronic AIC is more common than acute AIC. It is characterized by left ventricular systolic dysfunction accompanied by a reduced left ventricular ejection fraction (LVEF). The early symptoms of chronic AIC are often suppressed, but the clinical course tends to be irreversible. It can eventually progress to congestive heart failure or cardiomyopathy [34, 35]. In addition, cancer patients with previous cardiovascular disease (CVD) events or CVD risk factors are more likely to develop AIC.

At present, surveillance and assessment of AIC are mainly dependent on the measurement of myocardial injury-related biomarkers and imaging evaluation via echocardiography and magnetic resonance imaging (MRI) [36]. According to the definition from the Food and Drug Administration (FDA), AIC is interpreted as a decrease in LVEF by >20% when the baseline LVEF was normal or by >10% when the baseline LVEF was abnormal [37]; the left ventricular ejection fraction is recommended as the primary diagnostic criterion. Nevertheless, recent studies have shown that biomarkers such as high sensitivity Troponin I (hs-TnI), N-Terminal Pro-B-type Natriuretic Peptide (NT-proBNP), and B-type Natriuretic Peptide (BNP), as well as cardiac ultrasound parameters such as left ventricular end-diastolic volume (LVEDV) and global longitudinal strain (GLS), can compensate for the predictive capacity limitations of LVEF parameters alone. Combining multiple predictive markers can help identify patients with potential AIC [38–40].

Currently, targeted AIC therapy is lacking in terms of prevention and treatment. Nonpharmacological interventions include the following: (1) limiting the accumulated dose: a threshold dose of 550 mg/m^2^ for anthracycline and 900 mg/m^2^ for epirubicin [41]; (2) altering dosing strategy: slow continuous intravenous infusion (>6 h) which is preferable [42]; and (3) using liposomal anthracyclines [42, 43]. In pharmacotherapy, dexrazoxane is currently the only FDA-approved clinical drug for preventing AIC. Classic antiheart failure drugs, including angiotensin receptor blockers (ARB), angiotensin-converting enzyme inhibitors (ACEI), beta-blockers, and aldosterone antagonists, have also been shown to improve cardiac function during anthracycline treatment [44, 45]. Furthermore, statins can decrease the risk of cardiotoxicity by inhibiting topoisomerase II and the production of superoxide radicals [46]. However, there is no true gold standard for treating AIC among the above therapies. Exploration of AIC pathophysiological mechanisms is urgently needed to identify key therapeutic targets in AIC.

In terms of pathological mechanism, by binding both DNA and topoisomerase 2 (Top2) to form the ternary Top2-anthracycline-DNA cleavage complex, anthracycline inhibits the proliferation of cancer cells by impairing DNA replication and transcription, as well as inducing cell apoptosis [47]. Top2 is the major target of anthracycline is, especially the 2*α* isoform [48, 49]. However, binding to Top2*α* does not fully explain the cardiotoxicity of anthracycline, and a recent study found that the inhabitation of another Top2 isoform, Top2*β*, may be directly related to AIC [50]. Top2*β*, the only known type 2 topoisomerase found in cardiac mitochondria, can also combine with anthracycline to form anthracycline-DNA-topoisomerase 2*β* adducts [51]. This combination can significantly inhibit mitochondrial function and impair OXPHOS by targeting peroxisome proliferator-activated receptor gamma coactivator 1-alpha (PGC-1*α*)/PGC-1*β*, which is a key regulator of the mitochondrial biogenesis process [52].

In addition to the direct top2- (top2*β*-) dependent AIC mechanisms closely related to mitochondria, other top2*β*-independent mechanisms have emerged to explain AIC, emphasizing the central position of mitochondrial homeostasis in this process. Oxidative stress is the most widely accepted theory explaining the pathogenesis of AIC. It has been found that anthracyclines tend to accumulate primarily in cardiomyocyte mitochondria, especially the direct combination with cardiolipin in the IMM [53]. This combination promotes the impairment of complexes I and II in the mitochondrial ETC and excessive ROS production. ROS overproduction from impaired mitochondria can further damage mitochondrial DNA (mtDNA), lipids, and mitochondria-related proteins and depolarize the mitochondrial membrane, eventually leading to increased ROS overproduction and thereby resulting in a feedback loop. In addition, in impaired mitochondria, cardiolipin peroxidation induced by excessive ROS production can transform cytochrome c from an electron carrier into peroxidase, further oxidizing cardiolipin. Then, mitochondrial outer membrane permeabilization (MOMP) occurs, and proapoptotic factors such as cyto-c are released from intermembrane space (IMS) into the cytosol, which triggers cardiomyocyte apoptosis through caspase activation [54–56]. Moreover, morphological changes in mitochondria have been observed in doxorubicin-induced cardiotoxicity [57, 58].

Cellular chelatable and redox-active iron is defined as the labile iron pool (LIP), which also plays an important role in AIC onset and progression [59]. LIP can directly interact with anthracyclines and form iron–anthracyclines (ANTs) complexes that accelerate ROS production [60]. LIP can also react with superoxides (mostly H_2_O_2_) generated by anthracyclines and subsequently generate oxygen radicals including the highly toxic hydroxyl radical (^∙^OH) via the Fenton reaction [61]. Generated ^∙^OH molecules are strong oxidants with high redox potential, which induces nucleic acid mutations, lipid peroxidation, and so on. Anthracyclines have also been found to alter cellular iron homeostasis via various mechanisms. Anthracyclines can regulate maturation of mRNA that encodes transferrin receptors and ferritin by suppressing the activity of iron regulatory proteins 1 and 2 (IRP-1/2) [62]. Anthracyclines can also affect the cellular localization of iron so that less iron can be released from cellular storages like mitochondria [63]. The accumulation of iron in mitochondria has been related to the ferroptosis process, a type of iron-dependent cell death that is characterized by iron-dependent accumulation of lipid peroxides [64, 65]. The important role of ferroptosis in AIC was first reported by Fang et al., who found that ferroptosis was activated by Nrf2-Hmox1-mediated heme degradation and nonheme iron accumulation [66].

Nowadays, other than dexrazoxane, all the agents of ROS scavenging and iron chelating have not been recommended for the clinical treatment of AIC. However, a series of anti-inflammatory agents were found to exert cardioprotective effects in AIC, indicating that inflammation is an important potential mechanism of disease progression [67]. Moreover, it is known that oxidative stress and ROS overproduction are able to activate systemic inflammatory responses via induction of cytokine expression and complement activation, eventually leading to cell death [68]. Considering that anthracycline treatment may trigger severe multiorgan inflammation while exerting its antitumor effects, it is possible that anthracyclines can directly cause systemic toxicity via an inflammatory response in addition to the mediated contribution of ROS overproduction [69]. Zhu et al. also found that rhIL-1Ra, the natural antagonist of interleukin-1, can directly reverse increased proinflammatory cytokines levels after anthracycline treatment and alleviate associated myocardial damage [70].

Interestingly, in rat models and in clinical survivors of pediatric cancer chemotherapy, metabolic pathway alterations as well as mitochondrial inhibition persist even beyond the half-life of anthracyclines. This dose-dependently cumulative and progressive mitochondrial dysfunction has been referred to as “ANT cardiotoxicity memory” [71, 72]. There are several potential reasons that may underlie this phenomenon. Firstly, anthracycline treatment can result in cardiomyocyte loss by inducing cell death. Compared with adult survivors, this phenomenon is more common in pediatric patients due to the higher sensitivity of the apoptotic machinery in young myocardium [73]. Secondly, anthracycline treatment promotes oxidative modifications of mitochondrial DNA (mtDNA), suppressing the replication and expression processes. The dose accumulation and reduced transcriptional level of critical respiratory metabolic enzymes lead to decreases in mtDNA copy number and mitochondrial bioenergetic impairment, which presents as reduced ATP production and increased ROS production [74]. Moreover, continuous anthracycline chemotherapy may cause alterations in nuclear epigenetic regulation of critical redox and metabolic genes in cardiomyocytes. This may occur via direct interactions with DNA and histones or indirect regulation of epigenetic processes such as acetylation, methylation, and phosphorylation [75]. The anthracycline-induced epigenetic modifications ultimately lead to alterations in gene expression, which presents as disrupted myocardial metabolism and increased susceptibility to subsequent DOX chemotherapy [71].

In summary, excessive mitochondrial damage and disturbances in mitochondrial quality control failing to eliminate impaired mitochondria cause dysfunction or even cell death in cardiomyocytes. We believe that exploring the key role of MQC is vital to further understand the pathogenesis of AIC.

## 3. Mitochondrial Quality Control and AIC

### 3.1. Mitochondrial Dynamics

**Figure 1:**
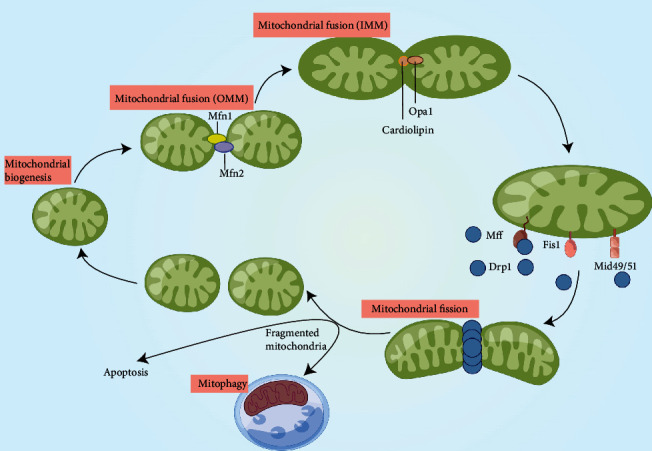
Regulatory mechanisms of mitochondrial dynamics. Mitochondria constantly undergo a dynamic cycle of fission and fusion. The fusion of two neighboring mitochondria depends on homo- and heterotypic interactions between Mfn 1/2 on the OMM. Following OMM fusion, IMM fusion is mediated by OPA1, which contains a cardiolipin- (CL-) binding site that interacts with the CL located on the IMM. During mitochondrial fission, phosphorylated Drp1 is recruited to the OMM via interaction with specific receptors, including Mff, Fis1, and Mid 49/51. The recruited Drp1 forms ring-like structures that constrict the organelle and produce two individual daughter mitochondria. During fission, impaired or dysfunctional components can be separated from the mitochondrial network and then degraded by mitophagy. However, mitochondrial fragmentation, resulting from excessive mitochondrial fission, can result in apoptosis by inducing mitochondrial outer membrane permeabilization (MOMP) and release of intermembrane space (IMS) proteins such as cytochrome c, mitofusins 1/2 (Mfn 1/2), optic atrophy protein 1 (OPA1), dynamin-related protein 1 (Drp1), mitochondrial fission factor (Mff), fission protein 1 (Fis1), and mitochondrial dynamic proteins of 49 and 51 kDa (Mid 49/51).

Benefiting from electron microscopy, the components of mitochondria are, from the outside to the inside, as follows: outer mitochondrial membrane (OMM), intermembrane space (IMS), inner mitochondrial membrane (IMM), and the mitochondrial matrix. The double membrane structural properties of the mitochondria determine that morphology is unlikely to remain unchanged. Recent studies have found that mitochondria are highly dynamic organelles that constantly cycle between fission and fusion events (Figure 1), dynamically adapting their size, density, number, distribution, and localization to the altering demands of the outside environment [76, 77]. The balance between mitochondrial fission and fusion is termed as mitochondrial dynamics [78]. The process of mitochondrial dynamics is essential for resource redistribution in the mitochondrial network and helps cardiomyocytes adapt to the stress environment.

Mitochondrial fission, the process of splitting intact mitochondria into two, is performed in three steps: (1) phosphorylation and activation of Drp1; (2) recruitment of Drp1 to the OMM via corresponding receptors, including fission protein 1 (Fis1), mitochondrial fission factor (Mff), and mitochondrial dynamic proteins of 49/51 kDa (MiD49/MiD51); and (3) by consuming GTP, the recruited Drp1 that oligomerizes on the OMM in the ring-like structure to divide into two individual organelles [79, 80]. Mitochondrial fission consists of two classes that differ in structure and function, termed midzone fission and peripheral fission, resulting in distinct daughter mitochondrial fates [81]. The former is primarily regulated by Mff and is designed to ensure substantial biogenesis capacity of the mitochondrial network in response to high energy demand under stress. However, the latter mainly acts in concert with mitophagy during impaired mitochondrial degradation. Peripheral fission induces the division of damaged components in mitochondria with low mitochondrial membrane potential (MMP), which are divided into smaller mitochondria and then cleaned from the mitochondrial network through mitophagy. If lacking the timely and innocuous removal of impaired mitochondrial content, substances such as Ca^2+^ or cytochrome c can be released into the cytosol, leading to irreversible cell death [81–83]. Therefore, the above two mitochondrial fission subtypes are both important aspects of MQC that function to maintain mitochondrial homeostasis.

Fusion is the tight connection between two individual mitochondria through the membrane merging process. The fusion of OMM and IMM is mediated by mitofusins and optic atrophy factor 1 (Opa1) respectively [84, 85]. The OMMs of two individual mitochondria are first connected by Mfn1 in the HR2 domains, followed by heterodimerization between Mfn1 and Mfn2 to promote OMM integration [86, 87]. In addition to serving as a regulator of mitochondrial cristae remodeling and maintenance process, Opa1 is a crucial regulatory protein involved in IMM fusion. Previous studies have indicated that excessive Opa1 downregulation by mitochondrial proteases, overlapping with m-AAA protease (OMA1) and i-AAA protease (YME1L), can induce drp1-independent mitochondrial fragmentation [88]. In other words, Opa1 not only acts as a classic fusion-related protein but also plays an important role in regulating fission events by altering its own expression under stress. Collectively, mitochondrial fusion appears to be a defensive response of the mitochondrial network in response to external stimuli. By integrating multiple resources such as proteins, metabolites, and mtDNA in two or more small mitochondria, the newly enlarged organelle can better adapt to the changing outer environment [89, 90].

Disturbed mitochondrial dynamics are closely related to a wide range of cardiovascular and metabolic diseases, such as diabetes, myocardial ischemia reperfusion injury (MIRI), and metabolic syndrome (MetS), which also includes AIC [91–93]. The imbalance between mitochondrial fission and fusion is found to be a centrally important mechanism that contributes to the progression of AIC. Mitochondrial fragmentation is found to be significantly increased in cardiomyocytes in the AIC animal model, which is accompanied by upregulated oxidative stress levels and ROS-induced apoptosis [94]. In neonatal rat cardiomyocytes treated with 0.86–1.72 *μ*mol/L doxorubicin for 1–24 h, it can be observed that the expression of fusion-related proteins like Mfn1/Mfn2/Opa1 decreased and Drp1 phosphorylation at serine 616 site specifically increased, indicating that the mitochondrial fission process is activated [90, 95]. This observation has also been confirmed in a study using postnatal rat cardiomyocytes and H9c2 cells treated with 5–10 *μ*M doxorubicin for 18–24 h, wherein the ratio of pSer616-Drp1/Drp1 remarkably increased [96, 97]. After treatment with the mitochondrial fission inhibitor Mdivi-1 (1 *μ*M for 30 min), a reduction in phosphorylation levels of Drp1^ser616^ and doxorubicin-induced myocyte apoptosis was noted, suggesting that excessive and uncontrolled mitochondrial fission may be a key aspect of AIC pathogenesis [98].

However, the role of mitochondrial fusion in AIC progression remains controversial. In rats treated weekly for 7 weeks with 2 mg/kg intraperitoneal (ip) injections of doxorubicin, fusion-related proteins like Mfn1, Mfn2, and Opa1 were found to be downregulated [99]. Although a series of prior studies have indicated a negative impact of anthracycline treatment on mitochondrial fusion, several other studies have suggested that activated fusion events play a key role in AIC progression. Another in vivo study in male C57BL/6 mice treated with a single dose of doxorubicin (10 mg/kg/ip) observed increased transcript levels of Mfn2, Opa1 (fusion), and Fis1 (fission) after 1.5 weeks of follow-up, but no significant changes in transcript levels of Drp1 [100]. We suspect that this discrepancy may be because of the differences in dosage and observation periods. Mitochondrial dynamics, especially fusion, play a compensatory protective role under acute administration of large dose of doxorubicin. With an increasing number of naïve daughter mitochondria induced by dox-enhanced mitochondrial fission, the mitochondrial network responds to excessive immature mitochondria through compensatory fusion events. Long-term administration of therapeutically effective doses may switch mitochondrial dynamics from a compensated to decompensated state and eventually result in enhanced fission but blunted fusion.

To date, the mechanism by which anthracycline regulates mitochondrial dynamic processes and its exact regulatory target remain unclear. Adenosine 5′-monophosphate- (AMP-) activated protein kinase (AMPK) is an intracellular serine/threonine kinase that functions as a central intracellular energy sensor and regulator of multiple metabolic pathways [101] and is also the major kinase regulating Drp1 protein. Anthracycline was found to significantly inhibit AMPK phosphorylation, promoting the phosphorylation of DRP1 at Ser637, which inhibited DRP1 oligomerization and suppressed fission [102]. Activation of the AMPK pathway with Shenmai injection (SMI) was shown to play a protective role in AIC by suppressing mitochondrial fission, and the SMI's promotion of Drp1 phosphorylation at Ser637 can be reversed by the intervention of AMPK inhibitor, Compound C [103]. Functioning as the major deacetylase in mitochondria, sirtuin 3 (SIRT3) is responsible for the deacetylation of the mitochondrial fusion-related protein Opa1, which can upregulate its activity and facilitate the protective influence of fusion on the mitochondrial network [104, 105]. AdSirt3 transfection of neonatal rat cardiomyocytes (NRCM) was observed to enhanced Opa1 acetylation and effectively alleviate apoptosis [106], suggesting that AMPK and SIRT3 may be the key targets of mitochondrial dynamic-related AIC treatment. In addition, miRNAs are considered to play an important regulatory role in anthracycline-induced mitochondrial dynamic disorders. An in vivo study using the NRCM model indicated that miR-532-3p, which directly targets the apoptosis repressor with the caspase recruitment domain (ARC), enhanced mitochondrial fission, and increased mitochondrial fragmentation [107]. In rat cardiomyocytes incubated with 1.0 *μ*M doxorubicin, the expression of Mfn1 was reduced and the fission process was activated compared to the control group. However, the intervention of miR-140 could rescue the downregulation of Mfn1 and alleviate doxorubicin-induced apoptosis and fission events [95]. Long noncoding RNAs (lncRNAs) can also participate in the regulation of mitochondrial dynamics. Cardiomyocyte mitochondrial dynamic-related lncRNA 1 (CMDL-1) was reported to be suppressed in doxorubicin-treated H9c2 cell, which inhibits doxorubicin-induced cardiomyocyte mitochondrial fission and apoptosis by targeting the fission-regulated protein Drp1 [108].

### 3.2. Mitophagy

Autophagy is considered an evolutionarily conserved mechanism that evolves to degrade intracellular cargo such as proteins or organelles in a lysosome-dependent manner. The proteins and organelles inside the autolysosomes are then converted into simple substances such as fatty acids, nucleic acids, and amino acids for recycling [109]. This process is accompanied by ATP production and plays a critical role in protein and organelle quality control systems. Autophagy is subdivided into three types according to its transport pathway into lysosomes: macroautophagy, microautophagy, and chaperone-mediated autophagy. Based on the nature of its function, autophagy can be further classified into two categories: nonselective and selective. Nonselective autophagy is designed to provide a nutrient substrate for cells in response to external stimuli, whereas selective autophagy targeting specific proteins or organelles serves to maintain intracellular homeostasis [110].

**Figure 2:**
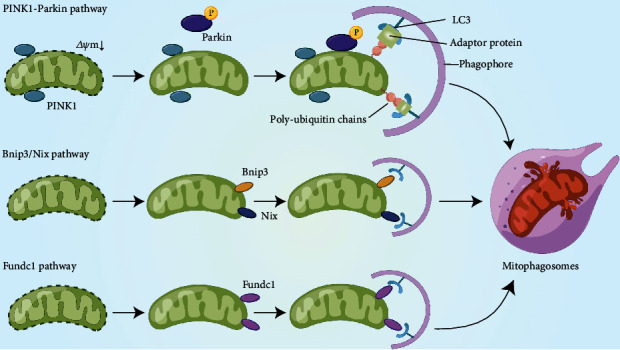
Regulatory mechanisms of mitophagy. PINK1 is degraded by presenilin-associated rhomboid-like (PARL) protease in the mitochondrial matrix under physiological conditions. When the MMP is impaired and decreased, PINK1 accumulates on the OMM, which recruits cytosolic Parkin to the impaired mitochondria and activates its E3 ligase activity through phosphorylation. The recruited Parkin catalyzes OMM protein ubiquitination and form poly-ubiquitin chains on OMM. These ubiquitinated proteins function as the recognition sequence domain of LC3 via interaction with adaptor proteins, such as p62/SQSTM1, leading to the transportation of damaged mitochondria into autophagosomes. Bnip3/Nix possess an LC3-interacting motif (LIR) can directly interact with LC3 on the phagophore independently of the adaptor protein p62/SQSTM1. Fundc1 is activated via phosphorylation under hypoxic conditions. Fundc1 interacts with LC3 directly through its LIR motif. Finally, the elongated isolation membrane encloses the impaired mitochondria and forms mitophagosomes, which then fuse with lysosomes for bulk degradation: PTEN-induced putative kinase 1 (PINK1), BCL2 and adenovirus E1B 19-kDa-interacting protein 3 (BNIP3), BNIP3-like (also known as BNIP3L) (Nix), and FUN14 Domain Containing 1 (FUNDC1).

Mitophagy, a type of selective autophagy essential for the elimination of dysfunctional mitochondria impaired by external stimuli, is vital for the regulation and sustentation of mitochondrial quality and quantity [111] (Figure 2). During this process, impaired mitochondria are first recognized by the isolation membrane (phagophore) and then encircled into these mitophagosomes, which are then transported into lysosomes for bulk degradation of organelles. Such a precise surveillance mechanism ensures that the intracellular mitochondrial network can be updated dynamically in response to changing environments. Mitophagy has also been found to play an important role in the progression of several diseases, such as Parkinson's disease (PD), diabetic kidney disease, and MIRI [112–115]. Furthermore, an increasing number of studies have suggested that mitophagy may exert a double-edge effect on disease progression [116–118]. Moderate mitophagy can remove depolarized mitochondria in a timely manner and avoid the release of proapoptotic molecules from these mitochondria and downstream amplification cascades. However, excessive mitophagy may lead to insufficient cellular energy supply and exacerbate cell death.

Although mitophagy is mediated and regulated by multiple pathways, it can be divided into two categories according to the difference in autophagosome recognition mechanisms: ubiquitin pathway-mediated mitophagy and receptor-dependent mitophagy. The former mainly refers to the PTEN-induced putative kinase 1- (PINK1-) Parkin pathway, while the latter includes BCL2 and adenovirus E1B 19-kDa-interacting protein 3 (BNIP3)/BNIP3-like- (BNIP3L, also known as Nix) mediated mitophagy and FUN14 Domain Containing 1- (FUNDC1-) mediated mitophagy [119].

#### 3.2.1. PINK1-Parkin-Mediated Mitophagy

The PINK1-Parkin pathway is the most representative of the Ubiquitin- (Ub-) dependent pathways and the most common type of mitophagy in mammalian cells [120]. PINK1 is a serine/threonine kinase with a hydrophobic mitochondrial localization signal at the amino terminus. Under physiological conditions, PINK1 is transported into mitochondria with a unique mito-targeting sequence and is degraded by the PARL protease in IMM [121]. However, degradation is disrupted in dysfunctional mitochondria with a decrease in the magnitude of membrane potential. In depolarized mitochondria, PINK1 degradation on the IMM is blocked owing to reduced PARL activity and is accompanied with accumulation of full-length PINK1 [121]. As a substrate of PINK1, Parkin can upregulate its E3 ligase activity via PINK1-induced phosphorylation, and the accumulated full-length PINK1 can recruit Parkin to the OMM of impaired mitochondria [122]. In addition, PINK1 can phosphorylate and activate Mfn2, which functions as the Parkin receptor of mitochondria [123]. This process also promotes the accumulation of Parkin in the OMM, where it ubiquitinates OMM-related proteins. These ubiquitinated proteins can serve as recognition sequence domains for LC3 that are recruited to the phagophore, and the interaction between autolysosomes and damaged mitochondria initiates mitophagy.

#### 3.2.2. BNIP3/Nix-Mediated Mitophagy

BNIP3 and Nix are BH3-only proteins in the Bcl-2 family. Their amino and carboxyl termini are located in the cytoplasm and OMM, respectively [124, 125]. BNIP3/Nix recruits cargo to autophagosomes by binding to impaired mitochondria via LIR motifs. Under hypoxic conditions, hypoxia-inducible factor 1-alpha (HIF-1*α*) can promote mitophagy by upregulating BNIP3 and Nix expression to remove damaged mitochondria timely [126]. Similar to FUNDC1, the interaction between BNIP3/Nix and LC3 was regulated by phosphorylation. The phosphorylation of BNIP3 at Ser17/Ser24 and the phosphorylation of Nix at Ser34/Ser35 facilitate mitophagy receptor-LC3 interactions [127]. But different from the classic PINK1-Parkin mitophagy pathway, the BNIP3/Nix-mediated mitophagy can lead to an unique kind of cell death—autophagic cell death, which can partly explain the biphasic effect of mitophagy on cell fate [124, 128].

The different mitophagy pathways are not isolated from one another but are interrelated via complex interaction mechanisms, especially reflected in the relationship between BNIP3/Nix and PINK1/Parkin pathways. On the one hand, BNIP3 can induce the Drp1-dependent mitochondrial fission and inhibit PINK1 degradation on OMM, resulting in the activated PINK1/Parkin pathway. On the other hand, Nix can also be ubiquitinated by Parkin and recruit the ubiquitous scaffold protein neighbor of BRCA1 gene 1 (NBR1) to impaired mitochondria, further promoting the receptor-dependent mitophagy process. Furthermore, interconnected regulation is observed not only in diverse mitophagy pathways but also in the relationship between mitophagy and mitochondrial dynamics. Mitochondrial fission activation is an essential prerequisite for mitophagy initiation. As described previously, mitophagy pathways, such as BNIP3/Nix or PINK1-Parkin, can also regulate the expression of dynamic-related proteins, such as Opa1 and Drp1, altering the mitochondrial dynamics equilibrium according to the immediate situation. Future studies should focus on mitochondrial dynamics as a novel intervention target in mitophagy-related AIC prevention and treatment.

#### 3.2.3. FUNDC1-Mediated Mitophagy

FUNDC1 is an OMM protein with an LIR at its amino terminus, whose activity can be regulated via phosphorylation. Under physiological conditions, the LIR of FUNDC1 is phosphorylated by casein kinase 2 (CK2) and Src kinase at Ser13 and Tyr18, respectively, inhibiting its interaction with LC3 and subsequent mitophagy. Nevertheless, the phosphorylation of CK2 and Src kinase is inhibited under hypoxic conditions. Moreover, phosphoglycerate mutase family member 5 (PGAM5) interacts with and dephosphorylates FUNDC1 at Ser13. Dephosphorylated FUNDC1 can interact with LC3 through LIR and initiate mitophagy. The interaction of FUNDC1 with LC3 can also be enhanced by phosphorylation of Ser17 by Unc-51-like autophagy activating kinase 1(ULK1). Thus, different regulatory modes of FUNDC1 phosphorylation which indirectly modulate the mitophagy seem to be promising therapeutic targets for AIC-related mitochondrial dysfunction.

FUNDC1 can also interact with dynamic-related proteins, such as Opa1 and Drp1, dynamically tuning the balance between mitochondrial fission and fusion to adapt to the actual mitophagy level. Physiologically, FUNDC1 localizes to the contact site linking the endoplasmic reticulum (ER) to the mitochondria and binds to the ER calnexin. It interacts with Opa1 to augment mitochondrial fusion. Under stress, FUNDC1 gets activated by dephosphorylation at the Ser13 site and the interaction with Opa1 is alleviated but enhanced with Drp1. Drp1 is recruited to mitochondria and initiates fission events, resulting in generation of excessive immature daughter mitochondria and further promoting mitophagy.

The role of the PINK1/Parkin pathway in AIC pathogenesis remains controversial. In transgenic mouse models with doxorubicin treatment five times for two weeks, it was found that p53 expression increased and the augmented interaction between cytosolic p53 and Parkin impaired the removal of damaged mitochondria by blocking Parkin mitochondrial translocation, thereby inhibiting mitophagy flux and enhancing the cardiotoxicity of doxorubicin. Knockdown of p53 or parkin overexpression was found to reverse this side effect [129]. Another in vitro study observed reduced expression of PINK1 and Parkin in 1 *μ*M doxorubicin-treated H9c2 cells [130]. These results echo well with the findings of in vivo experiments with a 4–20 mg/kg treatment dosage of doxorubicin [131–133], which indicated that the mitophagy process is repressed by doxorubicin treatment and results in excessive ROS production from impaired but not timely removal of mitochondria. However, Catanzaro et al. observed an increased level of Parkin-mediated mitophagy and Drp1-mediated mitochondrial fission in both H9c2 cells (750 nM Dox) and an AIC mouse model (15 mg/kg single dose of Dox) [94]. This phenotype can be attenuated by the downregulation of Parkin or Drp1 expression, indicating that AIC progression is associated with increased mitochondrial fragmentation and accelerated mitochondrial degradation by lysosomes. Moreover, superoxide accumulation as well as mitochondrial ultrastructural alterations were observed in dox-treated AC16 cells (15–250 nM Dox treatment for 24 h) in a concentration-dependent manner, and excessive mitophagy was found to be activated via the PINK1/Parkin pathway, accompanied by a significant increase in autophagosome markers such as LC3 and Beclin 1. Pharmacological interventions, such as mitophagy inhibition by mdivi-1 or scavenging mitochondrial superoxide by Mito-tempo, can suppress the overactivated PINK1/Parkin pathway by doxorubicin and rescue drug-induced cardiotoxicity [134].

The disagreement regarding the role of mitophagy in AIC across studies could be due to the differences in the dose/duration of drug administration. In a recent study, mitophagy flux was measured on days 2, 5, 8, and 14 following treatment with 18 mg/kg doxorubicin. Researchers found that the expression of PINK1/Parkin increased in the first week and subsequently decreased after two weeks of follow-up. Interestingly, the alterations in apoptosis level were found to be synchronous with the changes in mitophagy levels. The caspase-3 expression increased at one week, then gradually decreased to baseline for 2 weeks, suggesting that short-term mitophagy activation may be the direct mechanism of AIC [135]. Considering that mitophagy is a dynamic-adjusted process, the recovery of mitophagy level indicates the biological protective effects of compensatory mitophagy upregulation in the long term. In addition, mitochondria in cardiomyocytes can be divided into subsarcolemmal (SSM) and interfibrillar (IFM) mitochondrial subpopulations. SSM mitochondria mainly function to provide sufficient energy for membrane-related events, and IFM mitochondria serve as source for contractile activity-related energy and are more prone to release cytochrome c and apoptosis-inducing factors under stress [136]. These two subpopulations possess distinct biochemical properties and exhibit differential mitophagy receptor expression. A recent in vivo study compared the effects of doxorubicin on SSM and IFM mitochondria in an acute Dox-administered rat model. Although doxorubicin tends to be enriched in SSM mitochondria, IMF mitochondria exhibit a more dramatic mitophagy response to Dox treatment, including an increase in PINK1 and a decrease in p62/SQSTM1 [137]. The IMF mitochondria are more likely to be damaged by doxorubicin treatment. The subpopulation-specific differences in Dox-induced mitophagy levels may, in part, explain the divergence in the roles of the PINK1/Parkin mitophagy pathway between different groups.

Unlike the PINK1/Parkin pathway, translocation of BNIP3 to the mitochondria does not depend on membrane potential depolarization. BNIP33 can trigger the opening of the mitochondrial permeability transition pore (mPTP) and lead to downstream apoptosis events [138, 139]. BNIP3 also play an important role in the AIC. On the one hand, mPTP opening induced by BNIP3 activation can increase ROS production and inhibit interaction between respiratory chain complex IV subunit 1 (COX1) and uncoupling protein 3 (UCP3), leading to mitochondrial dysfunction and impaired mitochondrial membrane potential [140]. On the other hand, membrane potential depolarization activates the PINK1/Parkin pathway, which amplifies the mitophagy cascade. Dhingra et al. also found that knockdown of the Bnip3 gene can attenuate Dox-induced necrotic cell death [140]. A recent study reported that Sirt3 regulates Bnip3-mediated mitophagy following doxorubicin treatment. Upregulation of Sirt3 can effectively rescue excessive mitophagy from Bnip3 overactivation and promote the formation of COX1-UCP3 complex [141]. Another type of mitophagy, FUNDC1-mediated mitophagy, mainly plays a protective role in hypoxia-induced mitophagy. However, there are relatively few studies focusing on the role of FUNDC1-mediated mitophagy in AIC progression and treatment, which is worthy of further discussion.

### 3.3. Mitochondrial Biogenesis

**Figure 3:**
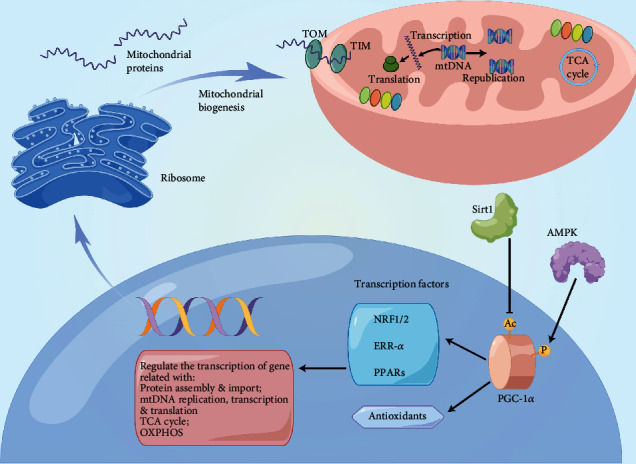
Regulation mechanism of mitochondrial biogenesis. PGC1*α* is the central regulator of mitochondrial biogenesis. The upstream regulatory mechanisms of PGC1*α* include AMPK phosphorylation and SIRT1 deacetylation. PGC1*α* modulates the expression of antioxidant proteins such as superoxide dismutase and glutathione peroxidase. More importantly, PGC1*α* activates downstream transcription factors including NRF1/2, ERR-*α*, and PPAR, which transactivate nuclear genes related to mitochondrial protein import and assembly, mtDNA transcription, replication and translation, TCA cycle, and OXPHOS. Genomic information is transcribed into mRNAs in the nucleus and then translated into proteins via cytosolic ribosomes. Nuclear gene-encoded mitochondrial proteins are transported into mitochondria via the TIM/TOM complex and are finally involved in the process of supplying cellular energy: peroxisome proliferator-activated receptor-*γ* coactivator 1*α* (PGC1*α*), AMP-activated protein kinase (AMPK), Sirtuin 1 (SIRT1), nuclear respiratory factor 1/2 (NRF1/2), estrogen-related receptor alpha (ERR-*α*), peroxisome proliferator-activated receptors (PPARs), translocase of the inner membrane (TIM), translocase of the outer membrane (TOM), mitochondrial DNA (mtDNA), tricarboxylic acid (TCA), and oxidative phosphorylation (OXPHOS).

Organelle biogenesis refers to the inheritance process of organelles during cell division [142, 143]. As the central bioenergy factory, mitochondrial biogenesis is not limited to the growth and division of mitochondria during normal cell cycle progression but can also occur in response to external stress or increase in cellular energy requirements during cell development [144]. Specifically, mitochondrial biogenesis is defined as the process by which cells generate new individual mitochondrial mass and replicate mtDNA (Figure 3).

Peroxisome proliferator-activated receptor *γ* (PPAR*γ*) coactivator 1*α* (PGC-1*α*) is the master regulator of mitochondrial biogenesis [145, 146]. It binds to and activates several nuclear transcription factors that transactivate nuclear genes for a series of biological events such as oxidative phosphorylation, substrate transportation, and mitochondrial protein import and assembly. These transcription factors include nuclear respiratory factor 1/2 (NRF1/2), estrogen-related receptor *α* (ERR-*α*), and PPARs [147]. NRF1/2 can further activate downstream transcription factor A mitochondrial (TFAM), which regulates mtDNA transcription and replication. Nuclear gene-encoded mitochondrial proteins are subsequently transported into the mitochondria via TIM/TOM complex [148, 149]. Moreover, PGC-1*α* can modulate the expression of antioxidant proteins such as superoxide dismutase and glutathione peroxidase. The upstream regulatory mechanism of PGC-1*α* is related to transcription factors that function on mitochondrial genes, such as the deacetylation of PGC-1*α* by SIRT1 and the phosphorylation of PGC-1*α* by AMPK or p38 MAPK pathway [150–152].

Mitochondrial biogenesis is a continuous dynamic process. Under physiological conditions, cardiomyocytes can dynamically regulate the biogenesis level via upstream modulators, such as the energy sensor AMPK, as per the immediate metabolism requirement. However, in pathological states, mitochondrial biogenesis is impaired, and the remaining mitochondrial network cannot guarantee an adequate energy supply. Currently, increasing emphasis is being placed on the role of mitochondrial biogenesis in AIC. Downregulation of Nrf2, PPAR*α*, and PGC-1*α* expression has been observed in mouse and rabbit AIC models, which is accompanied by ROS accumulation and apoptosis activation [153–155]. Cardiotoxicity is enhanced in Nrf2 knockout mice, possibly due to impaired autophagy and the accumulation of polyubiquitinated protein aggregates [156]. Another in vitro study with a functional 3D human multicell type cardiac system and human AC16 cells found that NRF2 gene inducers (bardoxolone methyl/isothiocyanate sulfuraphane) can mimic the protective effect comparable to dexrazoxane [157]. Recently, the adaptive response to low doses of doxorubicin has also been proven to be related to the activation of PGC-1*α* downstream proteins, such as NRF-1, MnSOD, UCP2, and COX1 [154].

### 3.4. Mitochondrial Protein Quality Control System

Human mitochondria consist of more than 1000 types of proteins, of which only the components of ETC (only 13 types of proteins) are encoded by mtDNA [89]. The vast majority of other mitochondrial proteins are encoded by nuclear DNA and then synthesized on cytosolic ribosomes to be delivered to the OMM or into the mitochondrial matrix via the TIM/TOM complexes. The newly synthesized mitochondrial proteins are first classified and then delivered to specific mitochondrial subcompartments according to their amino-terminal targeting signals, such as the mitochondrial targeting sequence (MTS) [158, 159]. MTS is designed to recognize and bind the TOM complex in mitochondrial protein translation and is degraded by presequence peptidases in the matrix after precursor proteins pass through OMM/IMM, completing the complex mitochondrial protein translocation process [160, 161]. In order to guarantee the synergistic exertion of physiological functions by these large number of proteins, mitochondria have evolved a precise set of protein quality control systems, including the mitochondrial molecular chaperones and proteases, and the mitochondrial unfolded protein response (UPRmt), which aid in supervising protein translation, import, folding, assembly, modification, and hydrolysis throughout the process.

#### 3.4.1. Mitochondrial Chaperones

Mitochondrial chaperones and proteases are central to maintaining mitochondrial protein homeostasis. After the precursor proteins, especially heat shock proteins (HSPs) such as HSP60/HSP10 and HSP70, enter the mitochondrial matrix, chaperones can assist in protein folding and assembly, as well as the refolding of denatured polypeptides to prevent the formation of aggregates [162, 163]. HSP60 interacts with its cochaperone HSP10 and bacterial homolog GroEL to form a barrel-like structure [164]. In vivo, the deletion of HSP60 in adult mouse cardiomyocytes led to the downregulation of approximately 20–30% in mitochondrial protein levels, triggering the early onset of mitochondrial unfolded protein response and downstream ROS overproduction, resulting in cardiomyocyte apoptosis and cardiac dysfunction [165].

Activated mitochondrial apoptosis signaling was observed in Dox-treated primary cardiomyocytes. Adenoviral overexpression of Hsp10 or Hsp60 can reverse the apoptosis of cardiomyocytes by upregulating antiapoptotic Bcl-xl and Bcl-2 and downregulating proapoptotic Bax. Moreover, Hsp10/Hsp60 can also function by suppressing the ubiquitination of Bcl-xl. The modulating function of posttranslational modifications in apoptosis-related proteins may explain the role of Hsp10/Hsp60 in the cardiac protection effect in AIC [166].

In addition, Liu et al. found that a single IP injection of doxorubicin at a dose of 15 mg/kg in an AIC mouse model exhibited a remarkable increase in circulating and myocardial Hsp70 expression. In the group following HSP70 neutralizing antibody treatment, considerable improvement in cardiac fibrosis levels and global cardiac function was observed. Mechanistically, anti-HSP70 antibody exerts cardioprotective effects mainly by suppressing the toll-like receptor 2-associated signaling cascade and modulating the nuclear factor-*κ*B (NF*κ*B) inflammatory pathway [167]. Physical activity, another key nonpharmacological intervention of AIC, has recently been found to function by increasing Hsp60 and glutathione (GSH) levels in cardiomyocytes [168].

#### 3.4.2. Mitochondrial Proteases

The activation of mitochondrial proteases is essential for the removal of unfolded or abnormal mitochondrial proteins. The proteins in the mitochondrial matrix are degraded mainly by three mitochondrial AAA proteases: the soluble mitochondrial Lon protease homolog (LonP1), mitochondrial ATP-dependent CLp protease (CLpP), and mitochondrial inner membrane-bound m-AAA protease [169]. There is also a group of proteases that exist in the intermembrane space responsible for the prompt clearance of mutated/misfolded proteins on IMM, including the membrane-bound ATP-dependent zinc metalloproteinase YME1L1, the soluble mitochondrial serine protease HTRA2, the mitochondrial metalloendopeptidase OMA1, and PARL [170]. Lon1 and CLpP are closely related to UPRmt, whereas other proteases, such as YME1L1, play an important role in other areas of mitochondrial proteostasis and dynamics. Reduced expression of the Lon protease homolog (LONP1) was found in a mouse model of pressure-overload heart failure, indicating decreased protein turnover and accumulation of abnormal proteins in impaired mitochondria [171]. In fact, a fine balance between the ATP-dependent metalloprotease (YME1L1) and the mitochondrial metalloendopeptidase (OMA1) is essential for regulating the cleavage of OPA1, which is the key regulator of mitochondrial fusion. Dysfunction of YME1L1 may lead to decreased levels of short OPA1 (S-OPA1) proteolytically cleaved by long OPA1 (L-OPA1) [172, 173]. Insufficient cleavage of OPA1 induces increased mitochondrial fragmentation, which is a pathological feature commonly found in myocardial dysfunction and heart failure [174].

#### 3.4.3. UPRmt

Misfolded protein accumulation in mitochondria is catastrophic as it can lead to OXPHOS impairment, decreased MMP, and enhanced release of proapoptosis signals [175, 176]. Mitochondria have evolved a set of mechanisms for adaptation to maintain proteostasis. Impaired proteins act as sensors of mitochondrial dysfunction to activate the reverse signaling transduction. By reverse transport of the defective proteostasis signal from mitochondria to the nucleus, the nucleus senses information and initiates the following transcription and translational events to upregulate the expression of mitochondrial chaperones and proteases, which can help in importing newly translated correctly folded functional proteins into mitochondria and degrade misfolded proteins in time. This adaptive reverse signal transduction from the mitochondria to the nucleus is termed as mitochondrial unfolded protein response (UPRmt) [177, 178]. UPRmt was first reported in *Caenorhabditis elegans* by Haynes et al. [179]. In mammals, the mitochondrial unfolded protein response mechanism consists of three main pathways: the ATF4/ATF5-CHOP, Sirt3-FOXO3a-SOD2, and eR*α*-NRF1-HTRA2 pathways [180]. These signaling axes exert synergistic effects by repressing proteotoxicity and rescuing mitochondrial proteostasis.

The beneficial role of UPRmt has been reported in a mouse model of myocardial infarction (MI) and pressure-overload heart failure. However, whether UPRmt plays a role in the prevention or treatment of AIC remains unclear. Further studies are required to explore how UPRmt dynamically adjusts to respond to anthracycline-induced stress during AIC progression. Furthermore, the development of UPRmt-related drugs is worthwhile.

## 4. Therapeutic Outlook

An in-depth understanding of mitochondrial toxicity in AIC will help in exploring novel AIC therapeutic strategies. However, applying mitochondria-targeting drug candidates to clinical AIC prevention and treatment without altering the antitumor effect requires further research. We provide an overview of the current pharmacological and nonpharmacological interventions for AIC and demonstrate their relationship with mitochondrial dysfunction. Furthermore, we summarized promising MQC-related therapies in clinical and preclinical models for future drug development.

### 4.1. Dexrazoxane

Currently, dexrazoxane is the only approved drug for protection against AIC by both the Food and Drug Administration (FDA) and European Medicines Agency (EMA) [181]. It is also the most widely studied drug for AIC. According to current guidelines, dexrazoxane is mainly used in patients with stage A heart failure (high risk of developing AIC pathology) [181]. Some researchers argue that dexrazoxane can also exert protective effects in patients with stage B heart failure [182]. Dexrazoxane functions as an iron chelator to attenuate the interaction between anthracyclines and nonheme iron, reducing the downstream oxidative stress levels [183]. In addition to its ability to chelate iron, the protective effect of dexrazoxane is partly attributed to the inhibition of the combination of Dox and the Top2*β*-DNA complex by tightly bridging the human Top2*α* and Top2*β* ATPase domain [184]. Furthermore, dexrazoxane can also inhibit poly ADP-ribose polymerase (PARP) to some extent, and the knockdown of PARP in the AIC model has been proven to prolong survival [185].

A recent study by Vijay et al. using the AIC mouse model focused on the Dox-induced transcriptional level alterations in mitochondria-related genes. Compared with the control group, the expression of 61 genes involved in energy metabolism and apoptosis was significantly altered by Dox treatment. However, the majority of these Dox-induced transcriptional changes were reversed by dexrazoxane pretreatment, which was accompanied with partly corrected plasma cardiac troponin T (cTnT) levels and cardiac function [186]. These results indicate that correction of mitochondrial dysfunction might be a potential therapeutic mechanism of dexrazoxane. It has also been found that dexrazoxane can alleviate the mitochondrial dysfunction in childhood acute lymphoblastic leukemia survivors treated with doxorubicin [187]. Considering that mtDNA encodes ETC components and can be compensatorily increased under mitochondrial impairment, another in vivo study observed that mtDNA copies per cell were increased in Dox-treated patients, and the copy number increase was reversed in patients treated with dexrazoxane at the same time, suggesting that mitochondrial function is preserved after dexrazoxane treatment [185]. This also indicates that mtDNA copy number can be developed in the future to screen patients with a high risk of AIC by monitoring the dynamic changes before and after Dox administration.

### 4.2. Novel Therapeutic Agents

The main advantage of dexrazoxane is that it can prevent doxorubicin-induced cardiac injury (such as increased troponin T levels), without interfering with antitumor activity of doxorubicin, making it a promising candidate for alleviating AIC [188]. Similar advantages have also been reported in recently developed endogenous and exogenous agents. Chromogranin A (CgA), a cardioprotective soluble protein present in the bloodstream and produced by both the neuroendocrine system and heart, was found to attenuate doxorubicin- (DOX-) related myocardial inflammation, apoptosis, oxidative stress, and ischemic injury. Moreover, the anticancer efficacy of DOX was not impaired in all investigated murine models [189]. Phenylalanine butyramide (FBA) is another novel protective agent against DOX cardiotoxicity, which prevents worsening of left ventricular dilatation and fibrosis. It also increases levels of atrial natriuretic peptide and brain natriuretic peptide resulting from doxorubicin therapy and increases the antitumor action of DOX. Similar to dexrazoxane, it mainly exerts its cardioprotective effects via decreasing oxidative stress and ameliorating DOX-induced mitochondrial dysfunction [190].

Furthermore, mitoTEMPO as well as other mitochondrial-selective antioxidants such as MitoQ show potential as therapies for AIC. These mitochondria-targeted ROS scavengers were found to reduce doxorubicin-induced cardiac injury by suppressing the PINK1/parkin mitophagy pathway, attenuating lipid peroxidation in heart mitochondria, restoring the expressions of cytochrome c oxidase (CcO) subunit II and Va and so on [134, 191, 192]. Considering their limited lipophilicity and accessibility to mitochondrial iron reservoirs, the protective effects of specific iron chelators such as deferiprone or deferoxamine have not been reported. However, ferrostatin-1, a ferroptosis inhibitor, has been proven to alleviate iron-mediated lipid peroxidation. In vivo experiments found that cardiotoxicity was significantly reduced in mice treated with both ferrostatin-1 and doxorubicin, indicating that ferroptosis inhibition is another promising approach in preventing AIC progression [66].

### 4.3. Chronic Physical Exercise

Chronic physical exercise is considered to be one of the most important nonpharmacological interventions for AIC. Various kinds of exercise, including treadmill training, swim training, and free wheel activity, have been shown to play a beneficial role in the treatment by maintaining mitochondrial homeostasis and attenuating oxidative stress. Exercise exerts beneficial effect through regulation of the MQC system from different aspects. In the context of mitochondrial dynamics, endurance treadmill (TM) training and voluntary free wheel (FW) activity were found to downregulate Drp1 and upregulate Mfn1/2 and Opa1 expression, which is accompanied by the downregulation of auto(mito)phagy signaling pathways such as PINK, p62, and Beclin/Bcl2 [193]. In terms of mitochondrial biogenesis and bioenergetics, Marques et al. reported that both TM and FW increase expression of PGC1*α* and complex I/II/III activities, but only TM can rescue the significant decrease in TFAM expression caused by subchronic Dox treatment [99].

### 4.4. MQC-Targeted Therapeutics

Increasing evidence indicates that the loss of MQC plays a key role in the development and evolution of AIC. In addition to the classic therapies discussed above, a wide variety of novel MQC-targeted agents are constantly being developed and tested to better prevent and treat AIC. The related MQC-targeted therapeutics are summarized in Table 1.

Inhibiting excessive mitochondrial fragmentation and recovering the fission/fusion balance have been suggested as key therapeutic strategies for AIC. LCZ696, a novel angiotensin receptor-neprilysin inhibitor (ARNi), was shown to significantly suppress Dox-induced mitochondrial dysfunction and morphology disturbance by inhibiting the phosphorylation of Drp1 ^ser616^. The Drp1-specific inhibitor, Midivi-1, has been shown to mimic the protective effects of LCZ696 [197]. The antiaging protein Klotho was also found to attenuate Dox-induced cardiotoxicity by inhibiting apoptosis and mitochondrial fission by downregulating Drp1 expression [194]. By upregulating fusion-related proteins such as Mfn-1 and Mfn-2, resveratrol treatment was shown to be sufficient to block Dox-induced left ventricular (LV) remodeling by maintaining mitochondrial homeostasis [196].

Although there is no consensus on the role of mitophagy in AIC currently, several agents have been reported to alleviate AIC by correcting Dox-induced excessive mitophagy in cardiomyocytes. The acetylcholinesterase inhibitor donepezil was found to attenuate mitochondrial injury by blocking mitophagy and autophagy flux in dox-treated rat cardiomyocytes, leading to decreased levels of inflammatory cytokines and ROS production and rescue receptor-interacting protein kinase 1- (RIP1-) mediated necroptosis [202]. In addition, liensinine, a novel mitophagy inhibitor, has also been shown to have a significant therapeutic effect on AIC, and the suppression of Dox-related mitophagy and phosphorylation of Drp1 at Ser616 may account for the therapeutic mechanisms of this bioactive compound [57].

Considering that mitochondrial biogenesis is substantially repressed during AIC progression, stimulation of mitochondrial biogenesis no doubt is another promising strategy for AIC treatment by assuring sufficient energy supply from the mitochondrial network. PGC-1*α* and Nrf-2 are central to the regulation of mitochondrial biogenesis, and the upstream regulatory mechanism focuses on AMPK and Sirt1. Therefore, the pharmacological activation of these genes has become an attractive issue in AIC treatment. Cyclovirobuxine D (CVB-D), an active steroid substance from *Buxus microphylla*, was shown to have a preventive effect in Dox-induced cardiac contractile dysfunction and rescued the impaired mitochondrial biogenesis during Dox treatment by preserving the expression of PGC-1*α* and mitochondrial DNA copy number [207]. In a Dox-treated mouse model, cotreatment with acacetin alleviated Dox-induced cardiac dysfunction and myocardial fibrosis. Nrf2/HO-1 and Sirt1/pAMPK signals were impaired during Dox treatment and were reversed by acacetin. Sirt1 knockdown abolished the protective effects observed in acacetin-treated mice, indicating that Sirt1-dependent mitochondrial biogenesis is a potential target for therapeutic intervention in AIC [211].

## 5. Discussion and Conclusions

To maintain mitochondrial homeostasis, the MQC system involves a series of adaptive responses including mitochondrial dynamics, biogenesis, mitophagy, and protein quality control. The loss of mitochondrial quality control may result in nonnormal phenotypes of mitochondrial shape and mass, degradation and regeneration, metabolism, and proteostasis.

**Figure 4:**
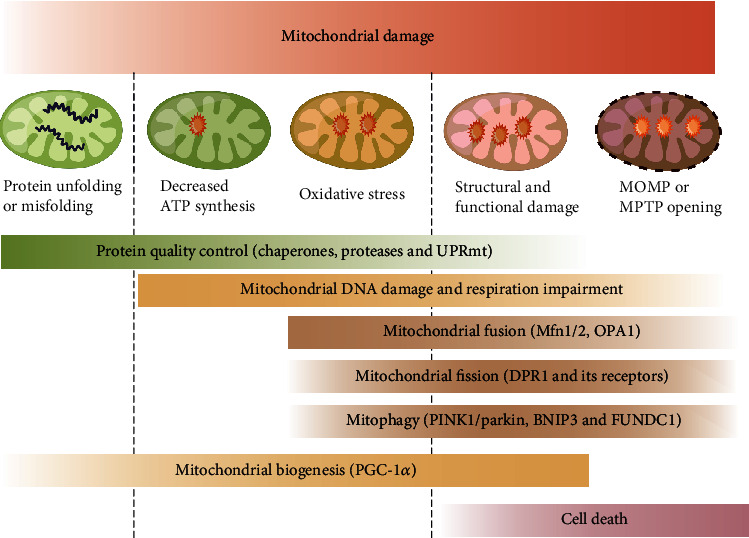
Mitochondrial quality control system. The mitochondrial quality control system includes both molecular and organelle quality control mechanisms. In the case of unfolded or misfolded proteins, protein quality control exerts actions mainly through chaperones which catalyze protein folding and ATP-dependent proteases which remove damaged proteins. When the protein quality control system is overloaded, the mitochondrial unfolded protein response (UPRmt) is activated. Signals from mitochondria are reversely transmitted to the nucleus to induce transcription of genes encoding mitochondrial chaperones, which subsequently enhances protein-folding capacity. Moderate mitochondrial stress is also accompanied by reduced ATP production and enhanced mitochondrial oxidative stress. DNA damage repair mechanisms are subsequently activated and function to repair damaged mitochondrial DNA. As mitochondrial injury becomes aggravated, severe mitochondrial stress triggers a series of alterations in mitochondrial structure and function, including mitochondrial fusion regulated by mitofusin 1 (Mfn1), mitofusin 2 (Mfn2), and optic atrophy 1 (OPA1); mitochondrial fission activated by cytosolic dynamin-1-like protein (Drp1); and its receptors. Daughter mitochondria resulting from segregation of damaged mitochondria are then targeted for degradation via mitophagy with specific adaptors such as Parkin, BCL-2/adenovirus E1B 19 kDa protein-interacting protein 3 (Bnip3), and FUN14 domain-containing 1 (Fundc1). Due to excessive mitochondrial fission, the reticular mitochondrial network is converted into fragmented mitochondria, which may reduce ATP production. Coupled with excessive mitophagy, the reduced mitochondrial mass results in impaired mitochondrial biogenesis, further leading to cellular bioenergetic crises. Daughter mitochondria can still be synthesized by the mitochondrial biogenesis process regulated by transcription factors like peroxisome proliferator-activated receptor-*γ* co-activator 1*α* (PGC-1*α*) but hindered production of nascent mitochondria eventually leads to cell death.

Doxorubicin binds with high affinity to cardiolipin which is located in the IMM, causing inefficient electron transfer across the ETC as well as ROS overproduction, disrupting mitochondrial homeostasis. The MQC system plays an important role in AIC pathogenesis (Figure 4). Based on the studies discussed in this review, it can be concluded that deregulated mitochondrial dynamics (excessive mitochondrial fission), uncontrolled mitophagy, impaired mitochondrial biogenesis, and failure to maintain mitochondrial proteostasis are characteristic features of AIC progression. Moreover, novel therapeutic strategies targeting the molecular mechanism and pathogenesis of AIC warrant further study in both preclinical animal models and clinical settings.

Additionally, we recognize that there are a number of important questions regarding MQC in the context of AIC that remains unanswered. First, although emerging evidence indicates that the mitochondrial protein quality control may play a role in the progression of AIC, the exact regulatory mechanism underlying mitochondrial chaperone/protease as well as UPRmt in the setting of AIC is still unclear. Further exploration is needed to provide a sufficient theoretical understanding of MQC-related treatment in AIC. Second, the mechanisms of the interaction between different MQC responses remain to be elucidated. Finally, an increasing number of studies have found several MQC-targeted agents at the stage of cell and animal AIC models. Most of these agents remain in the preclinical phase, and the related clinical evidence is lacking. Moreover, there is still no single drug or compound targeting the global MQC system has yet demonstrated clinical effectiveness in treating AIC.

## Figures and Tables

**Table 1:** MQC-targeted therapeutics in experimental models of AIC.

MQC-targeted agent	Molecular action	Key findings	Experimental objectives	References
*Mitochondrial dynamics*				
Klotho	Downregulator of DRP1	Alleviated mitochondrial fission and apoptosis	In vivo	[194]
Luteolin	Dephosphorylation of Drp1 ^Ser616^	Inhibited mitochondrial fission and oxidative stress	In vitro/in vivo	[195]
miRNA-532-3p	Targeting apoptosis repressor with caspase recruitment domain	Attenuated cardiomyocyte mitochondrial fission and apoptosis	In vivo	[107]
Shenmai injection	AMPK & PI3K/Akt/GSK-3*β* signaling pathway	Alleviated mitochondrial fragmentation and oxidative stress	In vitro/in vivo	[103]
Resveratrol	Activator of Mfn-1 and -2	Prevented left ventricular (LV) remodeling and reduced mitochondrial fragmentation	In vivo	[196]
LCZ696	Angiotensin receptor-neprilysin inhibitor (ARNi)	Attenuated Drp1-mediated mitochondrial dysfunction	In vitro/in vivo	[197]
Mdivi-1	Mitochondrial division inhibitor	Inhibited mitochondrial fission	In vitro/in vivo	[98]
Melatonin	PGC1-*α* and SIRT1 modulator	Decreased cell death and mitochondrial fission	In vitro/in vivo	[198]
Ciclosporin A	Inhibitor of mitochondrial membrane potential dissipation	Attenuated mitochondrial fragmentation and partially restored mitochondrial bioenergetics	In vivo	[100]
Honokiol	Activator of Sirtuin-3	Promoted mitochondrial fusion	In vitro/in vivo	[199]
Metformin	Activator of AMPK	Improved mitochondrial dynamics balance, biogenesis, and bioenergetics	In vitro/in vivo	[200, 201]
*Mitophagy*				
Heme oxygenase-1	A novel cardioprotective inducible enzyme	Rescued the changes in mitophagy mediators PINK1 and parkin	In vivo	[135]
Donepezil	Acetylcholinesterase inhibitor	Attenuated mitophagy, autophagy, and cardiomyocyte death	In vivo	[202]
Liensinine	A novel mitophagy inhibitor	Inhibited mitophagy and mitochondrial fragmentation	In vivo	[57]
Oseltamivir	Neuraminidase1 inhibitor	Inhibited the expression of Drp1 and PINK1 stabilization on mitochondria, attenuating excessive mitochondrial fission and mitophagy	In vivo	[203]
miRNA-22 knockout	SIRT-1/PGC-1*α* pathway	Alleviated mitophagy and mitochondrial biogenesis	In vitro/in vivo	[131]
*Mitochondrial biogenesis*				
CO/HO	Activator of Akt1/PKB and guanylate cyclase	Promoted mitochondrial biogenesis and alleviated apoptosis	In vitro/in vivo	[204]
Kirenol	Activator of PI3K/AKT and Nrf2 pathway	Rescued mitochondrial biogenesis and suppressed caspase-dependent apoptosis	In vitro	[205]
Nerolidol	Activator of Nrf2/MAPK signaling pathway	Mitigated oxidative stress, inflammation, and apoptosis	In vivo	[206]
Cyclovirobuxine D	Activator of PGC-1*α*/Nrf1 signaling pathway	Suppressed oxidative damage and mitochondrial biogenesis	In vivo	[207]
Metallothionein	Activator of PGC-1*α*/TFAM axis	Preserved mitochondrial biogenesis and MnSOD expression	In vivo	[208]
Yellow wine polyphenolic compounds (YWPC)	Nrf2/TGF-*β*/smad3 pathway	Mitigated oxidative stress, inflammatory response, and cardiac apoptosis	In vivo	[209]
Cannabidiol	Nonpsychoactive constituent of marijuana	Rescued the Dox-induced cardiac mitochondrial function and biogenesis impairment	In vivo	[210]
